# Supplementary material for: WordCommentsAnalyzer: A windows software tool for qualitative research
Source: F1000Res. 2018 Sep 4;7:536. Originally published 2018 May 3. [Version 2] doi: 10.12688/f1000research.14819.2 (PMC6137411; doi:10.12688/f1000research.14819.2)
Supplement: Supplementary file 3 [file f1000research-7-17614-s0002.tgz › b2a79496-4ada-47c4-9682-a99ce867836d.zip!/Eight Iranian Health Journals/Archives of Rehabilitation_12.docx]

© Archives of Rehabilitation (RJ). University of Social Welfare and Rehabilitation Sciences, Koodakyar Alley, Daneshjoo Blv., Evin, Tehran, Iran. This work is licensed under [CC BY-NC 4.0](https://creativecommons.org/licenses/by-nc/4.0/).

Volume & Issue: دوره 16 - شماره 2

Title: تجزیه و تحلیل مفهوم هندلینگ کاردرمانی در کودکان فلج مغزی: یک مطالعه هیبرید

http://rehabilitationj.uswr.ac.ir/article-1-1530-fa.html

هدف: هدف از این مطالعه تجزیه و تحلیل مفهوم هندلینگ کاردرمانی در کودکان فلج مغزی ، براساس تجربه کاردرمانگران بالینی و مدرسان کاردرمانی در جامعه ایرانی بود. روش بررسی: این مطالعه از نوع کیفی است که تجزیه و تحلیل مفهوم هندلینگ در کودکان فلج مغزی به روش هیبرید، در سه مرحله انجام شد. در مرحله نظری، مروری بر مقالات مرتبط موجود در پایگا ه ‌های اطلاعاتی معتبر تا سال 2014 صورت گرفت و مقالات ارزیابی و تحلیل شد . در مرحله کار در عرصه، نمونه گیری بصورت هدفمند و از طریق مصاحبه عمیق و نیمه‌ساختارمند با 6 کاردرمانگر بالینی، میزگرد بحث و گفتگوی تخصصی با حضور 11 کاردرمانگر و دو مشاهده در کلینیک صورت گرفت. سپس نتایج دست‌نویس شد و تحلیل محتوا صورت گرفت . در مرحله نهایی، تحلیل کلی از دو مرحله قبل به انجام رسید . یافته‌ها: پنج درونمایه اصلی برای مفهوم هندلینگ استخراج شد که عبارتند از: «مراقبت از کودک فلج مغزی»، «مدیریت درمان»، «درمان های دستی»، «آموزش مهارت‌های روزمره زندگی» و «حمل و جابه‌جایی». هم­چنین درونمایه‌های مربوط به ویژگی ‌ های هندلینگ براساس نظرات مشارکت‌کنندگان؛ کنترل، ایمنی، حرکت و وضعیت‌دهی شناسایی شدند . نتیجه‌گیری: به‌نظر می ‌رسد نتایج این پژوهش می‌تواند تا حدودی به واضح‌ تر شدن مفهوم هندلینگ کاردرمانی در کودکان فلج مغزی کمک نماید . همچنین با شناسایی فرایند عوامل تسهیل کننده، بازدارنده و مفهوم هندلینگ، مدرسین و کاردرمانگران بالینی خواهند توانست با تکیه بر دانش مبتنی بر یافته‌های علمی، فعالیت‌های درمانی و آموزشی خود را به‌گونه‌ای طراحی و اجرا کنند که شرایط لازم برای آموزش، یادگیری و اجرای صحیح هندلینگ در کاردرمانی فراهم آید.

Title: غربالگری تکامل حیطه فردی – اجتماعی کودکان 4 تا 60 ماهه با استفاده از آزمون غربالگری تکاملی دنور2 وپرسشنامه سنین و مراحل

http://rehabilitationj.uswr.ac.ir/article-1-1425-fa.html

هدف: این مطالعه با هدف بررسی وضعیت تکامل حیطه فردی–اجتماعی کودکان 60-4 ماهه شهر تهران با استفاده از دو آزمون غربالگری تکاملی دنور 2 و ASQ و بررسی میزان هم خوانی نتایج دو آزمون صورت گرفت. روش بررسی: 197 کودک 4 تا 60 ماهه که 93 نفر از آنان دختر و 104 نفر پسر بودند، مورد غربالگری تکاملی قرار گرفتند. نمونه گیری به روش نمونه گیری در دسترس انجام شد. یافته ها: در غربالگری انجام شده با دنور2 و ASQ به ترتیب %11 و %5/1 از کودکان مبتلا به تأخیر تکامل فردی-اجتماعی بودند. ضریب توافق کاپا بین دو آزمون 06/0 بود. نتیجه‌گیری: در این مطالعه از غربالگری تکامل حیطه فردی – اجتماعی کودکان تهرانی با استفاده از دو ابزار دنور2 و ASQ ، نتایج متفاوتی به دست آمده که نشانگر ضرورت تهیه و استانداردسازی یک آزمون تشخیصی طلایی است

Title: اثربخشی آموزش برنامه والدگری مثبت بر تعامل‌ مادر با کودک کم‌توان ذهنی

http://rehabilitationj.uswr.ac.ir/article-1-1624-fa.html

هدف: کم‌توانی ذهنی تمام جنبه‌های زندگی فرد را تحت تأثیر قرار می‌دهد در حالی که استفاده از برنامه‌های آموزشی والدگری با نتایج خوبی همراه است. هدف این پژوهش، بررسی تاثیر آموزش برنامه والدگری مثبت بر تعامل­ مادران با کودک کم‌توان ذهنی خویش بود. روش‌ بررسی: پژوهش حاضر، یک مطالعه شبه‌آزمایشی با طرح پیش‌آزمون- پس‌آزمون و گروه کنترل بود. 40 نفر از مادران دانش ­آموزان کم‌توان ذهنی که به روش نمونه‌گیری خوشه‌ای تصادفی از مدارس استثنایی شهرستان‌های تهران انتخاب شده بودند در این پژوهش شرکت داشتند. شرکت کنندگان به صورت تصادفی به دو گروه 20 نفری تقسیم شدند (یک گروه آزمایش و یک گروه کنترل). گروه آزمایش، برنامه آموزشی والدگری مثبت را در 10 جلسه دریافت کردند در حالی که به گروه کنترل این آموزش ارائه نشد. ابزار استفاده شده در این پژوهش مقیاس رابطه والد-کودک پیانتا (1994) بود. اطلاعات جمع‌آوری شده با استفاده از تحلیل کوواریانس چندمتغیری، در نرم‌افزار SPSS تحلیل شد. یافته‌ها: یافته‌ها نشان داد که گروه آزمایش و کنترل حداقل در یکی از خرده مقیاس‌های تعامل­ والد- کودک تفاوت معناداری داشتند.  نتیجه‌گیری: نتایج حاکی از آن بود که برنامه آموزشی والدگری مثبت سبب بهبود تعامل­ مادر با کودک کم‌توان ذهنی شده است. پیشنهاد می‌شود که متخصصان با اجرای برنامه‌ آموزشی والدگری مثبت، تعامل­ مادر با کودک کم‌توان ذهنی را ارتقاء بخشند.

Title: اثر تمرین اصلاحی فیدبکی بر والگوس زانو و فعالیت الکترومایوگرافی عضلات اندام تحتانی در اسکات یک پا

http://rehabilitationj.uswr.ac.ir/article-1-1547-fa.html

هدف: هدف از مطالعه حاضر بررسی اثر تمرین اصلاحی فیدبکی حین دویدن در مقابل آینه بر کینماتیک زانو و لگن در صفحه فرونتال و فعالیت عضلانی برخی از عضلات اندام تحتانی در اسکات یک پا (SLS) بود. روش بررسی: این مطالعه به روش شبه تجربی انجام شد که تعداد 23 آزمودنی زن سالم و فعال با میانگین سنی (2.43 ± 21.86) در دو گروه تمرینی و کنترل در این مطالعه شرکت کردند. گروه تمرینی شامل افرادی بود که در تست عملکردی SLS دارای زاویه افت لگن و والگوس زانوی بیشتری از مجموع میانگین ویک انحراف معیار جامعه بودند. قبل و بعد از 8 جلسه تمرین ، فعالیت عضلانی( RMS ) عضلات سرینی بزرگ، سرینی میانی، راست رانی، مایل داخلی، مایل خارجی، دوسر رانی و نیمه وتری، زاویه افت لگن و والگوس زانو در انتهای SLS ثبت شد. مقایسه متغیر های بین دو گروه توسط آزمون آماری تی مستقل و مقایسه در هر گروه توسط تی زوجی، با سطح معناداریی 0.05 صورت گرفت. یافته‌ها: قبل از تمرین نتایج تحلیل آماری تفاوت معناداری در زاویه افت لگن بین دو گروه نشان نداد (0/05&leP)، ولی زاویه والگوس زانو در گروه تجربی به شکل معناداری بیشتر از گروه کنترل بود(0/05&geP). هرچند فعالیت عضلانی (MVC %) در تمامی عضلات گروه تجربی بیشتر از گروه کنترل بود ولی به غیر از عضله دوسر رانی (0/05&geP) تفاوت معناداری بین دو گروه مشاهده نشد(0/05&leP). نتایج مقایسه قبل و بعد از تمرین در گروه تجربی تفاوت معناداری در کاهش والگوس زانو نشان نداد (0/05&leP)، با این وجود واالگوس زانو حدود 2 درجه کاهش یافت و علیرغم کاهش MVC % در تمامی عضلات تنها عضله راست رانی کاهش معناداری نشان داد (0/05&geP). در گروه کنترل نیز تفاوت معناداری در هیچ یک از متغیر ها مشاهده نشد(0/05&leP). نتیجه گیری: یافته ها نوعی ضعف عصبی عضلانی را در گروه تمرینی نشان میدهد که به دنبال تمرین تا حدی بهبود یافته است چراکه فعالیت و صرف انرژی کمتر عضلانی برای انجام حرکت خاص با کینماتیک مشابه نشان دهنده بهبود کنترل حرکتی یا ایجاد یادگیری است . به نظر میرسد تمرین اصلاحی مقابل آینه با فعال کردن نورون های آینه ای مسئول این یادگیری باشد . از طرفی فعالیت کمتر عضلانی در حرکات، کاهش خستگی را به دنبال دارد و از آن جا که بسیاری آسیب ها با خستگی ارتباط دارند می توان گفت با کاهش خستگی احتمالا بروز آسیب نیز کاهش می یابد . هرچند شواهدی وجود دارد که که این تمرینات باعث تغییرات مثبتی در والگوس زانو و فعالیت عصبی عضلانی اندام تحتانی می شود، ولی برای نتیجه گیری قطعی انجام مطالعات بیشتر مورد نیاز است.

Title: بررسی کیفیت زندگی مادرانِ کودکان اسپاینابیفیدا بر اساس معیار حرکتی Hoffer

http://rehabilitationj.uswr.ac.ir/article-1-1590-fa.html

هدف: مادرانِ کودکان مبتلا به اسپاینابیفیدا زمان بیشتری را صرف مراقبت از کودک خود می­کنند و احتمالا انجام چنین مراقبت­هایی، بسته به سطح عملکرد حرکتی کودک، می­تواند آسیب­هایی به سلامت جسمی و روانی مادران وارد کند. لذا هدف این مطالعه بررسی کیفیت زندگی مادران کودکان اسپاینابیفیدا، با توجه به معیار حرکتی Hoffer و با استفاده از پرسشنامه SF-36 می­باشد. روش بررسی: این مطالعه، یک مطالعه توصیفی –تحلیلی بوده و به صورت مقطعی اجرا گردید. جامعه آماری، کلیه مادران دارای کودک اسپاینابیفیدا 14-5/2 ساله شهر تهران و نمونه های در دسترس، مادران مراجعه کننده به کلینیک­های تحت نظارت دانشگاه علوم بهزیستی و توانبخشی بودند. از بین مادرانی که معیارهای ورود به مطالعه را دارا بودند 203 نفر در یک کلینیک مورد بررسی قرار گرفتند. ابزارهای مورد استفاده شامل معیار حرکتی Hoffer برای تعیین سطح عملکرد حرکتی کودکان اسپاینابیفیدا و پرسشنامه SF-36 برای تعیین کیفیت زندگی مادران بودند. تجزبه و تحلیل آماری داده‌ها با استفاده از آزمون t ، تحلیل پراکندگی چند متغیره یکراهه و چند راهه در نرم افزار SPSS ویرایش 18 صورت گرفت. یافته‌ها: تفاوت معنی داری در میانگین نمرات بعد جسمانی (P<0/01) و بعد روانی (P<0/01) کیفیت زندگی مادران در سطوح Hoffer کودک اسپاینابیفیدا دیده شد که مربوط به سطح یک Hoffer با سایر سطوح بود. تفاوت معنی داری در میانگین نمرات بعد جسمانی و بعد روانی کیفیت زندگی مادران در سطوح مختلف سنی و جنسیت کودک اسپاینابیفیدا وجود نداشت. تفاوت معنی داری در میانگین نمرات ابعاد جسمانی و روانی کیفیت زندگی مادران در انواع مختلف اسپاینابیفیدا وجود داشت که مربوط به نوع پنهان با انواع مننگوسل و میلومننگوسل بود (P<0/01) . نتیجه‌گیری: نوع اسپاینابیفیدا و سطح حرکتی Hoffer کودک، کیفیت زندگی مادر را در ابعاد جسمانی و روانی تحت تاثیر قرار می­دهد. می‌توان نتیجه گرفت که مادران دارای کودک اسپاینابیفیدا در کنار درمان کودکانشان، نیازمند توجه به کیفیت زندگی­شان هستند و باید خدماتی برای حمایت کیفیت زندگی آنان در ابعاد جسمانی وروانی ایجاد گردد.

Title: دورشدن از عملکرد مبتنی‌بر اکیوپیشن: تجربه کاردرمانگران ایرانی

http://rehabilitationj.uswr.ac.ir/article-1-1564-fa.html

هدف: ازابتدا، استفاده از اکیوپیشن به‌عنوان ابزار اصلی درمانی در الگوی کاردرمانی مطرح بوده است. با وجود تأکید اولیه پیشگامان کاردرمانی، هنوز کاردرمانگرها اهتمام جدی بر استفاده از اکیوپیشن در عملکرد بالینی خود ندارند؛ بنابراین مطالعه حاضر با هدف تبیین روش رایج ارائه خدمات کاردرمانی در بین کاردرمانگران ایرانی طراحی گردید. روش‌ بررسی: این مطالعه از نوع تحلیل محتوای کیفی-عرفی بود و در آن نمونه‌گیری به‌ شیوه هدفمند صورت گرفت. در این تحقیق 14 کاردرمانگر شرکت داشتند. برای جمع‌آوری اطلاعات از مصاحبه نیمه‌ساختاریافته استفاده شد و جمع‌آوری داده‌ها تا رسیدن به اشباع آنها ادامه یافت. مصاحبه‌ها با روش مقایسه مداوم و با استفاده از نرم‌افزار MAXQDA 2007 تحلیل گردید. یافته‌ها: تحلیل مصاحبه‌ها به ایجاد 66 کد اولیه منجر گردید که در تحلیل‌های بعدی به پیدایش شش زیرمضمون و سه مضمون اصلی انجامید: کاردرمانی مبتنی‌بر اکیوپیشن (رویکرد ارزیابی و برنامه درمانی مبتنی‌بر اکیوپیشن و شرایط سخت کاری برای کاردرمانگران اکیوپیشنی)، کاردرمانی مبتنی‌بر روش‌های درمانی (بی‌توجهی به عملکرد مبتنی‌بر اکیوپیشن، رویکرد ارزیابی و برنامه درمانی پایین به بالا و برنامه درمانی غیرمراجع‌محور) و کاردرمانی مبتنی‌بر تلفیق اکیوپیشن و ‌روش‌ها (ارزیابی و برنامه درمانی با رویکرد تلفیقی). نتیجه‌گیری: به ‌نظر می‌رسد که کاردرمانگران ایرانی از فلسفه پیدایشی حرفه خود یعنی عملکرد مبتنی‌بر اکیوپیشن- تا حدودی فاصله گرفته‌اند و به‌سمت استفاده از ‌روش‌های درمانی سوق پیدا کرده‌اند. از جمله دلایل احتمالی این موضوع می‌توان به فراهم‌نبودن زیرساخت‌های زمینه‌ای، عدم‌پذیرش مراجعین و مشکلات آموزشی اشاره کرد؛ بنابراین لازم است با برنامه‌ریزی و نظارت بیشتر از سوی سیاست‌گذاران، این کاستی‌ها برطرف و اجرای عملکرد مبتنی‌بر اکیوپیشن تسهیل گردد.

Title: تأثیر فعالیت‌های ورزشی بر مهارت‌های ادراکی-حرکتی کودکان سندرم داون چاق

http://rehabilitationj.uswr.ac.ir/article-1-1295-fa.html

هدف: هدف تحقیق حاضر، بررسی اثر هشت هفته تمرینات ورزشی منتخب بر مهارتهای ادراکی- حرکتی در دختران چاق هفت تا سیزده ساله سالم و سندرم داون بود. روش بررسی: در این مطالعه نیمه تجربی با گروه کنترل، 22 کودک چاق سندرم داون و 22 کودک چاق غیر سندرم داون که به صورت نمونه­ گیری در دسترس هدفمند انتخاب شده بودند به مدت 24 جلسه تمرینی در تمرینات منتخب ورزشی هدفمند شرکت کردند. همگی آزمودنی­ها، قبل و بعد از تمرینات، فرم کوتاه آزمون ادراکی-حرکتی برونینکز-ازرتسکی را انجام دادند. یافته­‌ها: نتایج نشان داد که هر دو گروه در مهارت­های حرکتی درشت پیشرفت معنی­داری (P<0/05) داشتند، اما در مهارت­های حرکتی ظریف این پیشرفت­ها معنی­دار نبودند. همچنین مقایسه میزان پیشرفت دو گروه نشان داد که کودکان سندرم داون چاق در هر دو گروه مهارت­های حرکتی درشت و ظریف به طور معنی­داری (P<0/05) پیشرفت بیشتری نسبت به کودکان غیر سندرم داون داشته­اند. نتیجه ­گیری: علی­رغم محدودیت­های فراوان ژنتیکی و محیطی اثر­گذار بر کودکان سندرم داون چاق، تمرینات ورزشی هدفمند مبتنی بر بازی و تفریح می تواند محدودیت­های ادراکی- حرکتی آنان را تا حد زیادی بهبود بخشد. لزوم تمرینات ادراکی- حرکتی زود هنگام در این کودکان بیشتر به چشم می­خورد.

Title: بررسی ارتباط بین حمایت اجتماعی و کیفیت زندگی در معلولین جسمی-حرکتی مراکز آموزشی رعد

http://rehabilitationj.uswr.ac.ir/article-1-1524-fa.html

هدف: حمایت اجتماعی از مهمترین عوامل پیش بینی کننده ی سلامت در افراد می باشد. هدف از مطالعه حاضر تعیین ارتباط بین حمایت اجتماعی و کیفیت زندگی معلولین جسمی-حرکتی مراکز آموزشی رعد است. روش بررسی: در این پژوهش که از نوع توصیفی- همبستگی می باشد، تعداد 195 نفر از معلولین جسمی-حرکتی 35-18 سال مراکز آموزشی رعد (رعد الغدیر، رعد شرق و رعد مرکزی)، به شیوه نمونه‌گیری طبقه ای تصادفی به نسبت سهمیه‌ای که هر یک از 3 مرکز به کل دارد و با روش تصادفی ساده تعداد مورد نظر انتخاب شد. پرسشنامه های حمایت اجتماعی وکس و کیفیت زندگی لنکشایر استفاده شده است. در تحلیل داده ها از آزمون های ضرایب همبستگی پیرسون و اسپیرمن و رگرسیون استفاده شد. یافته‌ها: نتایج بررسی‌ها نشان داد که بین خرده مقیاسهای حمایت اجتماعی (حمایت اجتماعی دوستان، خانواده و دیگران) با مؤلفه های روانی (آسایش روانی و خودپنداری) و مؤلفه های اجتماعی (روابط اجتماعی و روابط فامیلی) کیفیت زندگی در معلولین جسمی-حرکتی، رابطه مثبت معناداری وجود دارد ( P<0.01). نتیجه‌گیری: نتایج این مطالعه اهمیت حمایت اجتماعی را در کیفیت زندگی معلولین جسمی-حرکتی مورد تایید قرار می دهد لذا لازم است حمایت اجتماعی به عنوان یک تامین کننده قوی کیفیت زندگی، در طراحی مداخلات ارتقا سلامت معلولین مورد توجه قرار گیرد.
